# Nonionic Surfactants for the Cleaning of Works of Art: Insights on Acrylic Polymer Films Dewetting and Artificial Soil Removal

**DOI:** 10.1021/acsami.0c06425

**Published:** 2020-05-12

**Authors:** Michele Baglioni, Teresa Guaragnone, Rosangela Mastrangelo, Felipe Hidetomo Sekine, Taku Ogura, Piero Baglioni

**Affiliations:** † Department of Chemistry and CSGI, University of Florence, via della Lastruccia, 3, 50019 Sesto Fiorentino, Florence, Italy; ‡ NIKKOL GROUP Nikko Chemicals Co., Ltd., 1-4-8, Nihonbashi-Bakurocho, Chuo-ku, 103-0002 Tokyo, Japan; § NIKKOL GROUP Cosmos Technical Center Co., Ltd., 3-24-3 Hasune, Itabashi-ku, 174-0046 Tokyo, Japan; ∥ Research Institute for Science & Technology, Tokyo University of Science, 2641, Yamazaki, Noda-shi, Chiba 278-8510, Japan

**Keywords:** methoxy-pentadeca(oxyethylene) dodecanoate, pentadeca(oxyethylene) dodecyl ether, microemulsions, cleaning, conservation of cultural heritage, confocal laser scanning microscopy, fluorescence correlation spectroscopy, small-angle X-ray scattering

## Abstract

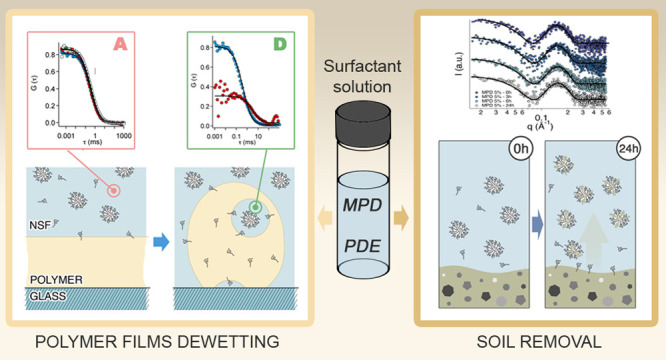

The use of nanostructured fluids (NSFs), that is, micellar solutions and microemulsions, in art conservation is often associated with cleaning purposes as the removal of polymeric coatings and/or soil from artistic surfaces. In both cases, the use of NSFs grants significant improvements over the use of traditional cleaning techniques that employ neat unconfined organic solvents, water, or aqueous solutions. The study of the nature and properties of surfactants present in NSF formulations is important to boost the effectiveness of these systems in applicative contexts and in the search of innovative and highly performing amphiphiles. This work reports on the methoxy-pentadeca(oxyethylene) dodecanoate (MPD) surfactant in two different NSFs, whose utilization in conservation of cultural heritage is new. Its effectiveness is compared to the conventional nonionic amphiphiles used in conservation practice, as pentadeca(oxyethylene) dodecyl ether, for the cleaning of poly(ethyl methacrylate/methyl acrylate) 70:30, p(EMA/MA), and artificially soiled surfaces. The mechanism, through which NSFs interact with polymeric coatings or soiled surfaces, was investigated by confocal laser scanning microscopy, fluorescence correlation spectroscopy, photographic observation, contact angle, surface tension measurements, and small-angle X-ray scattering. The results highlighted the superior MPD’s performance, both in inducing polymer removal and in detaching the soil from coated surfaces. At the microscale, the cleaning involves dewetting-like processes, where the polymer or the soil oily phase is detached from the surface and coalesce into separated droplets. This can be accounted by considering the different surface tensions and the different adsorption mechanisms of MPD with respect to ordinary nonionic surfactants (likely due to the methyl capping of the polar head chain and to the presence of the ester group between the hydrophilic and hydrophobic parts of the MPD surfactant molecule), showing how a tiny change in the surfactant architecture can lead to important differences in the cleaning capacity. Overall, this paper provides a detailed description of the mechanism and the kinetics involved in the NSFs cleaning process, opening new perspectives on simple formulations that are able to target at a specific substance to be removed. This is of utmost importance in the conservation of irreplaceable works of art.

## 1 Introduction

Nanostructured fluids (NSFs), such as micellar solutions and microemulsions, have been proposed as innovative cleaning systems in the field of conservation of cultural heritage, see Chelazzi et al.,^1^ Baglioni et al.,^2^ and references there in. Nowadays, they are an important part of the palette of the methodologies commonly used by conservators for the cleaning of works of art.^1−8^ NSFs used in art conservation are mostly related to two main cleaning issues: the removal of polymeric coatings^9−11^ (protective and consolidating agents, fixatives, adhesives, aged or fresh varnishes, graffiti, over-paintings, etc.) and the removal of soil^1,2,12−14^ (dust, particulate matter, grime, oily substances, sebum, wax stains, etc.). This last represents the most common of the interventions on artworks. In both cases, the use of NSFs grants significant improvements over the use of traditional cleaning methods, that is, the use of neat unconfined organic solvents, water, or aqueous solutions. The synergistic action of organic solvents and surfactants allows excellent cleaning performances, combined with a safe and controlled application. In fact, in a generic NSF, the organic solvent is confined in the water continuous phase and its amount is reduced to a few percentages, drastically lowering both the environmental impact of the methodology and the health risk for operators. Moreover, compared to unconfined organic solvents, NSFs are particularly effective for the removal of (hydrophobic) polymeric coatings. Different from organic solvents, which are chosen to dissolve a given polymer, NSFs are usually selected to be non-solvents for the polymer, in order to swell the film and detach it from the substrate surface through, depending on the polymer nature, a dewetting process.^15−18^ Dewetting is a well-known physical phenomenon defined as the spontaneous withdrawal of a film of fluid (i.e., from low viscosity liquids to highly viscous swollen polymers) from a surface and subsequent rearrangement in the form of separated droplets.^19−24^ The dewetting process of polymeric coatings from artistic surfaces induced by NSFs grants that polymer macromolecules are not spread into the work of art, as it would happen with neat unconfined organic solvents, resulting in an effective and controlled cleaning action. The nature of the organic solvents included in the NSF has a major role in the dewetting of polymers from solid surfaces, as they are selected to increase the mobility of polymer chains by swelling the film. Moreover, the surfactant nature is crucial to kinetically favor this process. In fact, the surfactant, lowering the polymer/solid interfacial tension, energetically favors the detachment of the film from the solid surface, and it was shown that a partial detachment of the polymer from the surface represents the first step of dewetting processes.^16−18^ Thus, amphiphile-based systems having low interfacial tension may be particularly effective as dewetting agents. Most recently, it was also observed that surfactants too have a role in increasing polymer chains mobility, making them the key components in NSFs for polymer removal.^17,18^

In many cases, works of art do not present polymeric coatings, but their visual aspect is compromised by the presence of soil/grime at the surface. Soil is composed of a variety of usually low molecular weight substances that accumulate on the surface of works of art as a result of ageing, unsuitable storage, or detrimental practices, from previous conservations, and so forth. A wide choice of cleaning methodologies is employed for soil removal, according to the specific needs of the given conservation case, spanning from the use of mechanical methods, to pure water, to the use of aqueous solutions of pH buffers, chelating agents, or surfactants, which may be applied by means of brushes, cotton swabs, poultices, thickeners, physical gels,^25−28^ or technologically more advanced solutions, such as highly retentive semi-interpenetrated or twin-chain polymer chemical gels, which grant the safest and most controllable cleaning action.^10,29,30^ Among the chemicals used for soil removal, surfactants certainly play a major role, and in particular when they are formulated as micellar solutions or microemulsions constitute the most effective tools available to conservators. Thus, nature and properties of surfactants are important to boost the effectiveness of NSFs in applicative contexts, and the search for innovative and highly performing amphiphiles is one of the main goals in the field of conservation of Cultural Heritage and in many practical applications in cosmetics, detergency, and so forth.

This work reports on the use of a relatively innovative surfactant, a methoxy-pentadeca(oxyethylene) dodecanoate (MPD),^31−35^ which is sometimes present in commercial detergents,^36−38^ but its cleaning mechanism is poorly understood and its utilization in conservation of cultural heritage is completely new, to the best of our knowledge. In particular, the effectiveness of MPD-based NSFs was studied and compared to the commonly employed PDE (C_12_EO_15_, pentadeca(oxyethylene) dodecyl ether)-based NSFs. In order to quantify the mechanism of action and the effectiveness of the cleaning systems, the MPD- and PDE-based NSFs were formulated to solve two conservative challenges: (i) polymer coatings removal and (ii) soil removal. In the first case, the removal of poly(ethyl methacrylate/methyl acrylate) 70:30, p(EMA/MA), commercially known as Paraloid B72, was studied on model systems as polymer-coated glass slides. The interaction mechanism between the polymer film and the cleaning fluid was investigated by means of confocal laser scanning microscopy (CLSM), fluorescence correlation spectroscopy (FCS), dynamic light scattering (DLS), and small-angle X-ray scattering (SAXS). Paraloid B72 is one of the most used polymers in conservation of cultural heritage,^39−42^ and it was widely used for a variety of different purposes and on different substrates. MPD- and PDE-based NSFs have been tested for soil removal from glass and polystyrene substrates coated with an artificial soil, prepared following standard procedures available in the literature,^43^ and characterized by means of visual and photographic observation, CLSM investigation, contact angle, surface tension measurements, and SAXS. Overall, MPD-NSFs were found to be superior over NSFs based on conventional nonionic surfactants.

## 2 Materials and Methods

### 2.1 Chemicals

C_11_(C=O)EO_15_–CH_3_, MPD (Nikko Chemicals, assay 99%), C_12_EO_15_, PDE (Nikko Chemicals, assay +99%), dodecyl dimethyl amine oxide (DDAO, Sigma-Aldrich, 30% aqueous solution), sodium dodecyl sulfate (SDS, Sigma-Aldrich, assay 99%), propylene carbonate (PC, Sigma-Aldrich, assay 99%), 2-butanone (MEK, Sigma-Aldrich, purity 99%), 2-butanol (BuOH, Sigma-Aldrich, assay >99%), ethyl acetate (EtAc, Sigma-Aldrich, ACS Reagents, assay ≥99.5%), and the fluorescent probes used for CLSM experiments, i.e., rhodamine 110 chloride, Nile red, coumarin 6 (Sigma-Aldrich, purity >98−99%), and Bodipy 558/568 C12 (4,4-difluoro-5-(2- thienyl)-4-bora-3a,4a-diaza-*s*-indacene-3-dodecanoic acid) (Thermo Fisher) were used without further purification. Water was purified with a Millipore Milli-Q gradient system (resistivity >18 MΩ cm). Carbon black, iron oxide (ochre), silica, kaolin, gelatin powder, Japanese paper (9.6 g/m^2^), poly(ethyl methacrylate/methyl acrylate) [p(EMA/MA)], Paraloid B72, pellets, and cellulose powder (Arbocel BC200, J. Rettenmaier & Sohne, Gmbh) were purchased from Zecchi, Florence. Soluble starch, cement, olive oil, mineral oil, and white spirit were commercially available and thus purchased in non-specialized stores.

### 2.2 Nanostructured fluids

The experiments reported in this study involved different NSFs. In particular, for the experiments on polymer (Paraloid B72) removal, four different formulations were selected, by combining the two surfactants MPD and PDE with two different organic solvents, PC and MEK, both partly miscible with water, and used as reference solvents in previous studies^16,17^ (see Tables S1 and S2). Besides MPD and PDE, an anionic (SDS) and a zwitterionic/cationic (DDAO) surfactant were used as reference amphiphiles.^44^ For soil removal experiments, micellar solutions of MPD and PDE were used, at two different surfactant concentrations, that is, 1 and 5% w/w.

### 2.3 Artificial Soil

The artificial soil mixture was prepared according to the standard formulation available in the literature^43^ and detailed in Supporting Information Table S3.

### 2.4 Sample Preparation

#### 2.4.1 CLSM Investigation on Polymer/NSF Interaction

For CLSM experiments on Paraloid B72/NSFs interaction, polymer films of about 2 μm thickness were prepared by spin-coating about 200 μL of a 10% w/w p(EMA/MA) solution in EtAc on coverglasses (2000 rpm, 120 s). The polymer films were stained with coumarin 6, co-dissolved with the polymer solution.

#### 2.4.2 Polymer Removal Tests on Glass Slides

Weighed 5 × 5 cm^2^ frosted glass slides were coated by drop-casting a 10% w/w p(EMA/MA), Paraloid B72, solution in EtAc, which was let drying until constant weight was reached. The average final amount of p(EMA/MA) on each glass slide was about 80 mg.

#### 2.4.3 CLSM Investigation on Soil/NSF Interaction

For CLSM experiments on soil/NSF interaction, glass slides were coated by drop-casting 150 μL of the artificial soil dispersion stained with Nile red 10^–6^ M, previously dissolved in white spirit. The samples were let completely drying for at least a week and then used for the experiments.

#### 2.4.4 Soil Removal Tests on Glass and Polystyrene Slides

Weighed 5 × 5 cm^2^ frosted glass and polystyrene slides were coated by drop-casting 1 mL of the artificial soil dispersion. The samples were let drying until constant weight was reached, and the final “dry” weight of the soil coating was about 2–3 mg/cm^2^, on average.

#### 2.4.5 FCS Investigation on Polymer/NSF Interaction

Paraloid B72 films were labeled by dissolving the hydrophobic dye coumarin 6 in the 10% w/w p(EMA/MA) solution in EtAc, to a final concentration of 1 mM ca. 2 μm thick films were prepared on glass slides through the same spin-coating procedure reported for CLSM experiments. FCS allows the tracking of fluorescent-labeled species diffusing in solution. Thus, the microemulsion droplets in solution were labeled by dissolving Bodipy in the NSFs to a final concentration of 10 nM. Bodipy is an amphiphilic dye with absorption and emission spectra well separated from the ones of coumarin 6.

### 2.5 Paraloid B72 Removal on Glass Slides

The study of the polymer removal was performed on frosted glass slides, prepared as reported in Section 2.4.2, using cellulose pulp poultices imbibed with the NSFs, and placing a sheet of Japanese paper between the poultices and the polymer film. The poultices were left interacting for 1.5 h, removed, and then the surface was gently rinsed with water to remove possible surfactant residues. After complete drying of the samples, the treated glass slides were weighed to obtain the % of removed polymer.

### 2.6 Soil Removal Tests on Glass and Polystyrene Slides

Soiled glass and polystyrene slides were immersed for 24 h in 40 mL of the following aqueous micellar solutions: MPD 1%, MPD 5%, PDE 1%, PDE 5% (w/w). During the experiments, the samples were not subjected to any mechanical action. At *t* = 0, 3, 6, and 24 h, the immersed samples were photographed and the cleaning fluid in contact with the soil layer was sampled by taking small amounts of liquid, which was subsequently investigated by SAXS measurements, in order to follow the possible NSF structural evolution during the interaction with soil. After 24 h, samples were taken out from the NSFs and tilted with care, in order to check for the residual adhesion of the soil coating to the glass/polystyrene surface.

### 2.7 Confocal Laser Scanning Microscopy

Confocal Microscopy experiments were performed on a Leica TCS SP8 confocal microscope (Leica Microsystems GmbH, Wetzlar, Germany) equipped with a 63× water immersion objective. Rhodamine 110 chloride and coumarin 6 were excited with the 488 nm laser line of an argon laser, while Nile red was excited with a DPSS solid state laser at 561 nm. The emission of the dyes was acquired with two PMTs in the range 498–530 and 571–630 nm, respectively. CLSM experiments were performed to monitor the interaction of the polymer films or soil with different NSFs, as detailed in Section 2.2.

#### 2.7.1 Paraloid B72/NSF Interaction

Unlabeled liquid phase (200 μL) were left in contact with the coumarin 6-stained Paraloid B72-coated coverglass, and the morphological variations of the polymeric film were monitored over time, up to 20 min.

#### 2.7.2 Soil/NSFs Interaction

Liquid phase (200 μL) labeled with rhodamine 110 chloride were left in contact with the Nile red-stained soiled coverglass, and the morphological variations of the soil coating were monitored over time, up to 10 min.

### 2.8 Contact Angle Measurements

Surfactant adsorption was indirectly evaluated by measuring the contact angle of 5 μL of Milli-Q water droplets on soiled glass slides with a Rame-Hart model 190 CA Goniometer. Three samples were analyzed, that is, pristine soil-coated glass slide and two soil-coated glass slides immersed for 1 min in a 1% w/w MPD and PDE solution, respectively. The equilibrium contact angle was measured in at least five different areas, and the average value and standard deviation were evaluated.

### 2.9 Surface Tension Measurements

Surface tension values of MPD and PDE aqueous solutions were determined with a K100 Tensiometer (Krüss, GmbH, Hamburg, Germany). The surface tension was measured at different concentrations by adding a concentrated stock solution of surfactant in water to a known volume of water (40 mL). Surface tension measurements were carried out with a platinum plate, and for each concentration, the average of ten readings was taken after attaining the equilibrium.

### 2.10 Small-Angle X-ray Scattering

SAXS measurements were performed with a HECUS S3-MICRO SWAXS-camera, equipped with a Hecus System 3 2D-point collimator (min divergence 0.4 × 0.9 mrad^2^), and two position sensitive detectors (PSD-50M) consisting of 1024 channels with a width of 54 μm. The *K*_α_ radiation (λ = 1.542 Å) emitted by a Cu anode from the Oxford 50 W microfocus source with customized FOX-3D single-bounce multilayer point focusing optics (Xenocs, Grenoble) was used, while the *K*_β_ line was removed using a multilayer filter. The voltage was generated by the GeniX system (Xenocs, Grenoble). The sample-to-detector distance was 26.9 cm. The volume between the sample and the detector was kept under vacuum during the measurements to minimize the scattering from the atmosphere. The camera was calibrated in the small-angle region using silver behenate (*d* = 58.38 Å). Scattering curves were obtained in the *q*-range between 0.008 and 0.5 Å^–1^. The temperature control was set to 25 °C. Samples were contained in 2 mm thick quartz capillary tubes sealed with hot-melting glue. Scattering curves were corrected for the empty capillary contribution considering the relative transmission factors. Desmearing of the SAXS curves was not necessary thanks to the focusing system. The fitting model adopted is described in detail in the Supporting Information file.

### 2.11 Fluorescence Correlation Spectroscopy

FCS measurements were performed with a Leica TCS SP8 confocal microscope (Leica Microsystems GmbH, Wetzlar, Germany) equipped with a PicoQuant FCS modulus (PicoQuant, Berlin, Germany). A water immersion objective 63×/1.2 W (Zeiss) was used. The evolution of the structure of the fluorescent-labeled film during the interaction with the NSFs was followed by confocal imaging, exciting the green dye coumarin 6 with the 488 nm laser line, and collecting the emitted signal with a PMT in the range 498–530 nm, as reported in the Confocal Laser Scanning Microscopy section. Once the polymer film structure was stabilized (i.e., no fast rearrangements were occurring) the diffusion of Bodipy, located at the microemulsion droplet interfaces, was monitored through FCS. The dye was excited with the DPSS 561 laser (561 nm), while the fluorescence intensity was acquired using a hybrid SMD detector in the 571–630 nm range. Freshly-prepared samples (water/solvents, water/surfactants and the four NSFs labeled with 10 nM Bodipy) were, at first, analyzed before the interaction with the polymer film by pouring the solutions in the appropriate sample-holder (Lab-Tek Chambered #1.0 Borosilicate Coverglass System, Nalge Nunc International, Rochester, NY, USA). Then, 200 μL of the labeled solutions were poured on the polymer-coated glass slides. During the liquid–polymer interaction, different areas were probed through FCS measurements. Depending on the sample, the diffusion of Bodipy was measured either in the liquid-filled cavities formed at the polymer/glass interface (for MEK-based NSFs), or in the cavities found inside the dewetted polymer (for PC-based NSFs), and in the bulk liquid on the top of the polymer film, after 20 min of interaction (for all the systems). Measurements were performed at 25 °C. More details on the data analysis are reported in the Supporting Information file (FCS data analysis).

### 2.12 Dynamic Light Scattering

DLS measurements were performed on a Brookhaven Instruments apparatus (BI 9000AT correlator and BI 200 SM goniometer) equipped with a EMI 9863B/350 photomultiplier. The 633 nm He-Ne laser was used to avoid light absorption by the Bodipy labeled systems. Measurements on the simple water-surfactant systems and on the NFSs were carried out at 90 and 25 °C. The signal was collected performing 8 min measurements, and the diffusion coefficients were obtained either from a second-order cumulant analysis or by the weighted average of the values obtained by the CONTIN algorithm.^45^ In the second case, the values of diffusion coefficients were obtained as weighted average of the most recurrent components (components accounting for less than 8% of the population were not considered). All data shown are the average of three repetitions, with relative standard deviations.

## 3 Results and Discussion

This study focuses on the mechanism and kinetics of MPD cleaning and detergent properties in the context of cultural heritage conservation. As reported above, two main conservative issues are the scope of this study, that is, polymer removal and soil removal. Besides testing the efficacy of proposed NSFs based on MPD surfactant, our aim was to understand the interaction between this surfactant and the materials to be removed because in art conservation the value of the works of art impose very specific, controlled, and performing cleaning, without any possible damage to the original works. To this aim, all the experiments on MPD behavior and performances were compared to those obtained replacing MPD surfactant with its alcohol ethoxylate homologue, PDE.

Recently, a thorough study by Sato et al.^46^ showed that the physico-chemical behaviors of MPD and PDE are significantly different, despite their similar molecular architecture. In particular, phase behavior in water and the polar head hydration are different for the two surfactants. This leads to a different excluded volume for the micelles and different effective micellar volume fraction for the two systems. These findings partly explain the experimental evidence that MPD possesses better cleaning efficiency than PDE, especially in low mechanical conditions, that is, when micellar solutions are kept in contact with soiling materials without stirring. In particular, the removal of oleic acid from fabrics by PDE and MPD micellar solutions was studied by means of several techniques, and it was found that at the equilibrium state both surfactants have almost the same emulsification and solubilization power toward oleic acid, while, before the system is equilibrated, PDE is preferentially adsorbed onto oleic acid coatings in the form of lamellar structures. On the contrary, MPD is less efficiently adsorbed onto the soil surface and tends to solubilize the oil in the hydrophobic core of micelles.^47^

### 3.1 Polymer Film Removal

**Figure 1:**
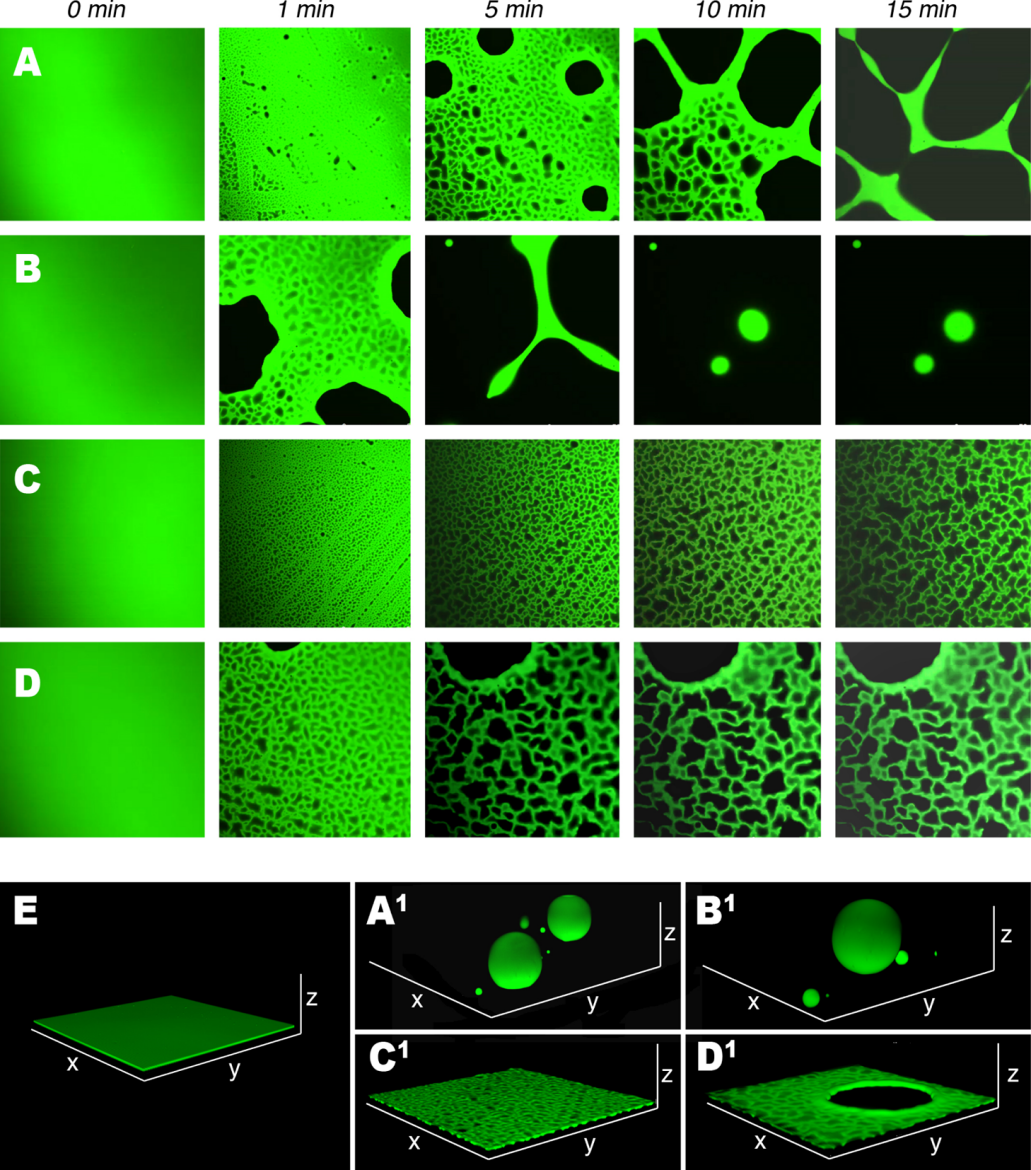
CLSM results on p(EMA/MA) interacting with (A) H_2_O/PC/PDE, (B) H_2_O/PC/MPD, (C) H_2_O/MEK/PDE, and (D) H_2_O/MEK/MPD. (E) 3D reconstruction of the polymer film before the interaction with NSFs, (A^1^–D^1^) 3D reconstructions of the polymer after 20 min of interaction with (A^1^) H_2_O/PC/PDE, (B^1^) H_2_O/PC/MPD, (C^1^) H_2_O/MEK/PDE, and (D^1^) H_2_O/MEK/MPD, which clarify the morphology of the film at the end of the experiments. The bottom side of each CLSM frame is 150 μm long.

As already stated, polymer film removal with NSFs usually involves dewetting. From a thermodynamic point of view, the tendency of a film to dewet from a surface is described by the spreading coefficient, *S*, which accounts for the energetic balance of the system. In the case of a polymer film laid on a glass surface and immersed in a liquid, *S* is expressed as follows^48^
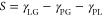
where γ_LG_ is the interfacial tension between the glass and the liquid, γ_PG_ is the interfacial tension between the glass and the polymer, and γ_PL_ is the interfacial tension between the polymer and the liquid. In total wetting regime (*S* > 0), films are always stable and dewetting does not occur. On the other hand, when a fluid (or a polymer, considered as a fluid in this context) is only partly wetting (*S* < 0), films are unstable or metastable and dewetting is thermodynamically favored below a critical thickness *h*_c_, which for most substances is in the range of millimeters. Even in these last conditions, dewetting does not necessarily take place. In fact, it can be inhibited by a kinetic factor, that is, an energy barrier has to be overcome in order to induce the process. For thin films (thickness, *h* < 100 nm), this energy barrier is usually low and the film is unstable. This instability generates capillary waves through the film, and when their fluctuation exceeds the film thickness *h*, the film itself spontaneously breaks down into separated droplets according to a mechanism termed spinodal dewetting.^19^ Thick films (*h* > 100 nm), on the other hand, are metastable. A 2 μm-thick hydrophobic polymer film laid on a hydrophilic surface, such as glass, as in the case of the experiments here reported, is a good example of metastable system, where dewetting would be thermodynamically favored but kinetically inhibited because of the low mobility of entangled macromolecular chains in the film. Whenever this mobility is enhanced, the film becomes unstable and dewetting occurs. Enhanced chain mobility can be induced essentially in two distinct ways: (i) the film is heated at a temperature higher than its glass transition temperature, *T*_g_(49) and (ii) the film is exposed to some organic solvents, which swell the polymer, lowering its *T*_g_ below room temperature.^22,50^ The experiments shown in Figure 1 belong to this latter case. The figure reports the results of CLSM investigation on the interaction of 2 μm thick p(EMA/MA) films, deposited on glass slides, with four different NSFs based on two different organic solvents, PC and MEK, and the two surfactants object of this study, MPD and PDE. PC and MEK were selected as the NSF organic solvents because it was recently found that they show a different behavior in inducing p(EMA/MA) dewetting, and, in particular, PC is more efficient than MEK.^16,17^

The interaction process was monitored at the polymer/glass interface. Figure 1 shows the morphological evolution of continuous polymeric films (visible in green). Upon interacting with the NSFs, some dark areas appear in the confocal plane, meaning that the polymer is no longer present in those areas. The observation of the film along the *z*-axis (here not reported) shows that the dark areas are not holes that go through the whole film thickness; instead, they are liquid-filled cavities that form as the polymer is locally detached and lifted from the solid surface. The rims of polymer remaining onto the glass surface draw a characteristic shape termed Voronoi pattern or tessellation.^19^ As the cavities grow and coalesce, they become weaker and the film eventually breaks with the nucleation of holes, according to a well-known and described mechanism for the dewetting of thick films,^19^ and as the glass is exposed to the bulk liquid phase, the polymer withdraws from the surface in the form of thick rims, which again describe a Voronoi pattern but on a larger scale. Complete dewetting is reached when polymer rims are also disrupted and swollen polymer globular droplets form.

It was found that the two PC-based NSFs are able to completely dewet the polymer from the glass, while in the case of MEK-based NSFs, no complete dewetting was observed after 20 min of interaction (see Figure 1). Interestingly, considering the time for the dewetting onsets for NSFs based on the same solvent (compare the series of Figure 1A,B), MPD is more efficient than PDE in inducing polymer dewetting. After only 5 min, the polymer interacting with the H_2_O/PC/MPD system is almost completely dewetted, while at the same time, the film interacting with the H_2_O/PC/PDE system showed just a few 20–30 μm large holes in an otherwise continuous polymer film. The same trend could be observed in the MEK-based systems. Figure 1C,D clearly shows that the interaction process is boosted by the presence of MPD. The difference in the dewetting process can be explained in view of the mechanism through which dewetting takes place in its early stages. When the polymer is locally detached from the solid surface, a portion of the polymer/glass interface is “destroyed”, while new interfacial regions are formed between the polymer/liquid and the glass/liquid phases, with an overall increase of the total interfacial area of the system. It was found that the main role of surfactants in this process is reducing the energy costs related to the formation of this intermediate state, by lowering the interfacial tension.^17^ Therefore, the interfacial tension of both surfactants was measured over a wide concentration range, from well above the cmc of the surfactants to more than 100 times lower (see Figure S5). It was found that the surface tension of the MPD micellar solution, γ_MPD_ ≈ 34.5 N/m, is lower than that of PDE, γ_PDE_ ≈ 37.5 N/m, which is in agreement with a kinetically boosted dewetting process for this surfactant. In addition, it can be hypothesized that the presence of the methyl capping at the end of the polyoxyethylene chain of MPD confers to this surfactant an increased hydrophobicity and thus a higher capability of penetrating into the p(EMA/MA) film, with a consequent enhancement of polymer chains mobility. Overall, these factors account for the better performances of MPD over PDE.

**Figure 2:**
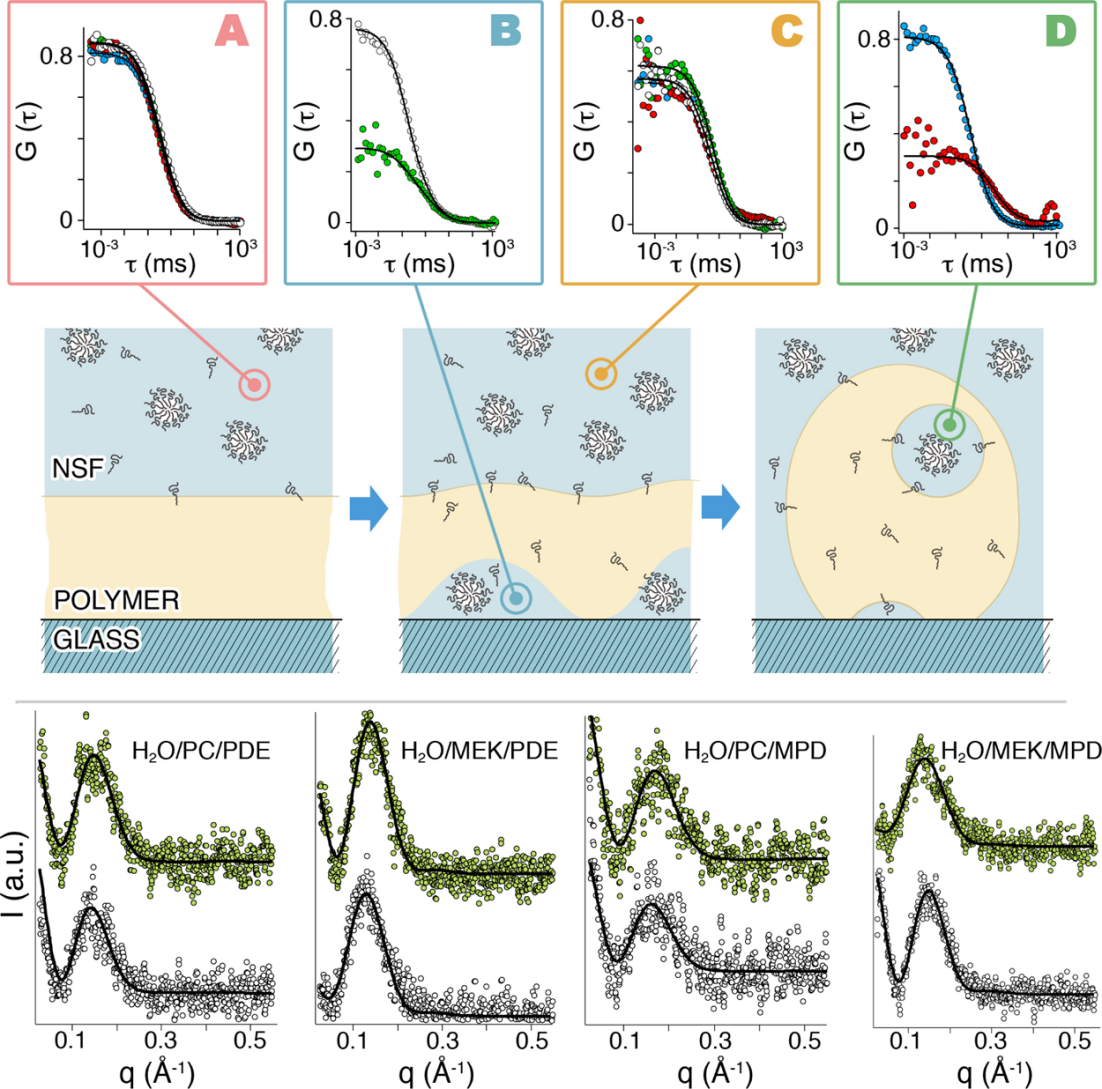
Cartoon illustrates the behavior of the NSFs droplets. After the local detachment of the polymer from the glass surface, the complex fluid droplets penetrate through the polymer and reach liquid-filled cavities. The top boxes report the FCS curves taken at *t* = 0 min (A), at time *t* = 5–15 min (B,D) and after 20 min of incubation (C) of the polymer with the four different NSFs; H_2_O/PC/PDE (blue circles), H_2_O/PC/MPD (red circles), H_2_O/MEK/PDE (white circles), and H_2_O/MEK/MPD (green circles). The best fittings are shown as solid black lines. (Bottom) SAXS profiles of the four NSFs before (white circles) and after (green circles) 20 min of incubation of interaction with the polymer. The best fittings are shown as solid black lines. The curves have been arbitrarily offset for sake of clarity.

In order to get a detailed picture of the polymer dewetting process, induced by MPD- and PDE-based NSFs, the diffusion and the evolution of the droplets during the film/liquid interaction were investigated by means of FCS and SAXS measurements. The main results of these experiments are reported in Figure 2.

For confocal experiments, micellar solutions (H_2_O/PDE 5% and H_2_O/MPD 5%) were labeled with Bodipy, as described in the Materials and Methods section. In order to determine if the Bodipy dye addition affects the micelles, the diffusion of micellar species in labeled and unlabeled systems was measured through DLS. The results show that micelles’ diameter does not significantly change after labeling (the *D* values obtained by the cumulant analysis are reported in Table 1).

**Table 1:** Average Diffusion Coefficients, *D* (μm^2^/s), Obtained by DLS Analysis

system	*D* (μm^2^/s) for unlabeled sample	*D* (μm^2^/s) for Bodipy-labeled sample
H_2_O/PDE	85 ± 3^a^	81 ± 2^a^
H_2_O/MPD	88 ± 6^a^	86 ± 3^a^
H_2_O/MEK/PDE	85 ± 11^b^	87 ± 10^b^
H_2_O/PC/PDE	72 ± 5^b^	75 ± 15^b^
H_2_O/MEK/MPD	73 ± 5^a^	87 ± 6^b^
H_2_O/PC/MPD	77 ± 2^a^	78 ± 2^b^

a Values and standard deviations obtained by cumulant analysis.

b Weighed averages of the most recurrent *D* values obtained by CONTIN algorithm, with standard deviation.

PC or MEK addition to the unlabeled or labeled micellar systems produces small changes of the micellar diffusion coefficients, indicating that micelle size is only slightly affected. The diffusion coefficients reported in Table 1 were obtained as weighed average of the most recurrent *D* values obtained from the CONTIN analysis.

The analysis of the light scattering data by the CONTIN algorithm on labeled and unlabeled systems returns an average diffusion coefficient of 80 μm^2^/s that was used as “guess value” for the FCS data analysis. SAXS measurements performed on the four NSFs before and after 20 min of interaction with the polymer were also used as an input for FCS analyses. Figure 2 (bottom) shows the fitted scattering profiles of all the investigated samples, while Table 2 reports the main fitting parameters. SAXS analysis shows that the size, shape, and polydispersity are poorly affected by the interaction of MPD and PDE-based NSFs with the Paraloid B72 film, suggesting that up to 20 min of application, the NSFs do not solubilize the p(EMA/MA) polymer film.

**Table 2:** SAXS Fitting Parameters for the NSFs, Measured before and after the Interaction with the p(EMA/MA) Film^a^

system	fitting parameter	before interaction	after 20 min interaction
H_2_O/MEK/MPD	*r*_c_ (Å)	16.2 ± 0.1	15.2 ± 1.6
	*t* (Å)	15.3 ± 0.4	15.6 ± 3.4
	PDI	0.12 ± 0.01	0.15 ± 0.01
H_2_O/PC/MPD	*r*_c_ (Å)	11.5 ± 0.2	12.0 ± 3.6
	*t* (Å)	19.7 ± 0.7	17.0 ± 6.5
	PDI	0.15 ± 0.01	0.15 ± 0.01
H_2_O/MEK/PDE	*r*_c_ (Å)	17.3 ± 0.1	16.8 ± 0.8
	*t* (Å)	16.0 ± 0.5	16.2 ± 1.6
	PDI	0.14 ± 0.01	0.12 ± 0.01
H_2_O/PC/PDE	*r*_c_ (Å)	13.2 ± 0.1	13.8 ± 2.5
	*t* (Å)	22.1 ± 0.6	18.7 ± 4.7
	PDI	0.15 ± 0.01	0.15 ± 0.01

a PDI is the polydispersity index; *r*_c_ is the average core radius; *t* is the shell thickness.

The confocal analysis of the cleaning process indicated that dewetting was, as expected, the main process for the polymer removal. FCS was used to shed light on the cleaning mechanism in the “dewetting-like” polymer removal. The autocorrelation functions, *G*(*t*), obtained by FCS measurements have been analyzed considering two-components decays (see Supporting Information, FCS data analysis) and using as initial guess the parameters obtained from DLS and SAXS data analysis. The diffusion of the Bodipy-labeled NSFs was measured through FCS, before and during the interaction with the polymer film, in different sample regions, as shown in Figure 2. Data on the diffusion of the droplets forming the NSFs disperse phase were collected both inside the liquid-filled cavities that form in the swollen polymer (i.e., at the polymer/glass interface for MEK-based systems, inside the dewetted polymer droplets for PC-based systems), and in the bulk liquid on top of the film, after 20 min of interaction.

The results are shown in Figure 2A–D, and the calculated diffusion coefficient, *D*, are reported in Tables 3 and 4.

**Table 3:** Diffusion Coefficient Values (μm^2^/s) and Percentage of *D*_1_ Component in the Total Decay, Obtained Through the Fitting of FCS Curves of MEK-Based NSFs^a^

NSF	*D*_bulk_ (*t* = 0 min)	*D*_cav_ (*t* = 5 min)	*D*_cav_ (*t* = 12 min)	*D*_bulk_ (*t* = 20 min)
H_2_O/MEK/PDE	*D*_1_ = 80 (40%)	*D*_1_ = 80 (80%)	*D*_1_ = 80 (80%)	*D*_1_ = 80 (40%)
	*D*_2_ = 17 ± 2	*D*_2_ = 0.4 ± 0.2	*D*_2_ = 6 ± 2	*D*_2_ = 16 ± 5
H_2_O/MEK/MPD	*D*_1_ = 80 (50%)	*D*_1_ = 80 (70%)	*D*_1_ = 80 (60%)	*D*_1_ = 80 (50%)
	*D*_2_ = 17 ± 3	*D*_2_ = 7 ± 3	*D*_2_ = 6 ± 3	*D*_2_ = 6 ± 2

a FCS was performed into liquid-filled cavities trapped inside the dewetted polymer (Cav) and in the solution on the top of the film *D*_bulk_. Error for *D*_1_ component is about 10%.

**Table 4:** Diffusion Coefficient Values (μm^2^/s) and Percentage of *D*_1_ Component in the Total Decay, Obtained through Fitting of FCS Curves of PC-Based NSFs^a^

NSFs with PC	*D*_bulk_ (*t* = 0 min)	*D*_cav_ (*t* = 10 min)	*D*_bulk_ (*t* = 20 min)
H_2_O/PC/PDE	*D*_1_ = 80 (50%)	*D*_1_ = 33 ± 2 (99%)	*D*_1_ = 80 (50%)
	*D*_2_ = 19 ± 4	*D*_2_ = 0.01	*D*_2_ = 13 ± 4
H_2_O/PC/MPD	*D*_1_ = 80 (60%)	*D*_1_ = 5 ± 2 (90%)	*D*_1_ = 64 ± 40 (40%)
	*D*_2_ = 18 ± 4	*D*_2_ = 0.1	*D*_2_ = 4

a FCS was performed into liquid-filled cavities trapped inside the dewetted polymer (Cav) and in the solution on the top of the film *D*_bulk_. Error for *D*_1_ component is about 10%.

The composition and aggregates’ size in the bulk MEK-based NSFs remains almost the same before and after the interaction with the polymer film, except for the slow component *D*_2-bulk_ in the H_2_O/MEK/MPD system, which decreases after the interaction. However, the most remarkable features of these systems lie in the description of the diffusive behavior of labeled species in the NSF confined into the polymer cavities that form at the polymer/glass interface. The main result is that according to measured diffusion coefficients, NSF droplets are able to penetrate inside these cavities (see *D*_1-cav_ values in Table 3). Thus, the swollen film is somehow permeable to the passage of either micellized or monomeric surfactant. In fact, data show that the polymer film is more easily penetrated by the smaller droplets, diffusing at 80 μm^2^/s.

The values of *D*_2-cav_ for both MEK- and PC-based NSFs suggest the presence of micelles/microemulsion droplets–polymer interactions inside the cavities formed at the polymer/glass interface. This can be explained either as diffusion coefficient of NSF droplets being slowed down by the interaction with the polymer walls of the cavity or as droplet growth due to the solubilization of low-molecular weight polymer chains extracted from the swollen polymer. Apart from this similarity, the two surfactants show a different behavior.

In the case of the H_2_O/MEK/PDE system, the NSF inside the confined cavities never reaches the diffusion coefficient of bulk NSF on top of the polymer film (i.e., *D*_2-cav_ ≠ *D*_2-bulk_), suggesting that the interaction with the NFS only slightly alters the polymer film permeability, and the polymer film acts as a sort of “molecular sieve”, where only smaller aggregates, probably swollen surfactant micelles, are able to reach the liquid-filled cavities at the polymer/glass interface.

On the other hand, the H_2_O/MEK/MPD NSF shows an evolution with time, that is, the NSF confined into the cavities at the polymer/glass interface eventually reaches the diffusion coefficient of bulk NSF located above the polymer film. The decrease of *D*_2_ values at *t* = 20 min seems to be mainly ascribable to the extraction and solubilization of low-molecular weight polymer chains into micelles/microemulsion droplets.

The analysis of PC-based NSFs was more complicated in view of the fact that the polymer is completely and relatively quickly dewetted from the glass surface. It was not possible to perform any FCS measurements into the cavities that form at the polymer/glass interface, as they evolved too fast. Conversely, it was possible to measure the diffusion of labeled species inside the liquid-filled cavities that were found trapped into the large droplets of swollen polymer (see the right image of the cartoon in Figure 2), which remain onto the glass surface at the end of the dewetting process.

The H_2_O/PC/PDE NSF composition before and after the interaction is almost the same. The diffusive species detected inside the cavities are, in part, strongly interacting with the polymer walls (*D*_2-cav_ = 0.01), while the *D*_1-cav_ value is probably an average of faster and slower diffusing species. In fact, before the rearrangement of the polymer in the form of large droplets, the NSF penetrates the film and is confined in the cavities at the glass/polymer interface, see panel D in Figure 2.

On the other side, the H_2_O/PC/MPD NSF significantly changes during the interaction with the polymer. Slow-diffusing species can be found both in the cavities confined into dewetted polymer droplets and in the liquid on top of the film. These data confirm that, as in the case of H_2_O/MEK/MPD, micelles/microemulsion droplets are probably able to extract and dissolve some low-molecular polymer chains present inside the polymer film, and this effect is boosted by the co-presence of both the most effective solvent, PC, and surfactant, MPD.

In conclusion, the different NSFs show different mechanisms that depend both on the organic solvent and surfactant forming the NSF. Considering the obtained results, we challenged the NSFs to the removal of Paraloid B72 polymer films to real cases, that is, thickness of several μm. We compared and quantify the performances of four different NSFs having the same composition (see Table S2, in Supporting Information) but different surfactant. Besides MPD and PDE, two additional surfactants (SDS, DDAO) were selected and used as reference.

Figure S6 in Supporting Information reports the outcome of the cleaning tests with the % of polymer removal obtained via gravimetric measurements. All the selected NSFs resulted highly effective for Paraloid B72 removal from glass slides, yielding an average removal of about 75% of the polymer after a single application of 1.5 h. Some slight differences could be spotted among different NSFs, for example, DDAO is the less effective of the tested surfactants, with a removal of 69 ± 3%, and MPD, with a removal of 78 ± 1%, was the most effective.

### 3.2 Soil Removal

Soil removal is a very complex subject because of the number of variables mainly linked to the heterogeneous composition of soil. The chemical nature of soiling materials and artworks constituents, the micro-morphology of the surface, and possible surface/soil interactions are only some of the factors that might change from one case to another.

In the present study, glass and polystyrene slides were used as specimen for the experiments. They were coated with very thick layers (∼10–20 μm) of artificial soil; this amount of soil is not easily encountered on real artworks surfaces, where the soil layer is usually less than 1–2 μm thick. Therefore, these experimental conditions have been chosen to amplify possible differences in the behavior of different NSFs involved with the cleaning process. Furthermore, the composition of the artificial soil used in the present work (see Table S3) is very complex, including different materials, ranging from oils (i.e., mixtures of more or less hydrophobic molecules, such as alkanes, fatty acids, fatty acid esters and triglycerides) to more hydrophilic polymeric materials (i.e., gelatin and starch) and to an inert mineral fraction composed of carbon black, iron oxide, kaolin, and silica. This makes the understanding of the interaction process between the NSFs and the soil coating more complex. Most likely, a synergistic combination of concomitant physical phenomena occurs; however, this system is closer to real cases.

Several studies dealing with artificial or real soil removal in the context of conservation of cultural heritage are reported in the literature.^14,51−56^ However, to the best of our knowledge, this paper reports for the first time an insight on the interaction mechanism occurring when a surfactant-based NSF is in contact with a soiled surface.

**Figure 3:**
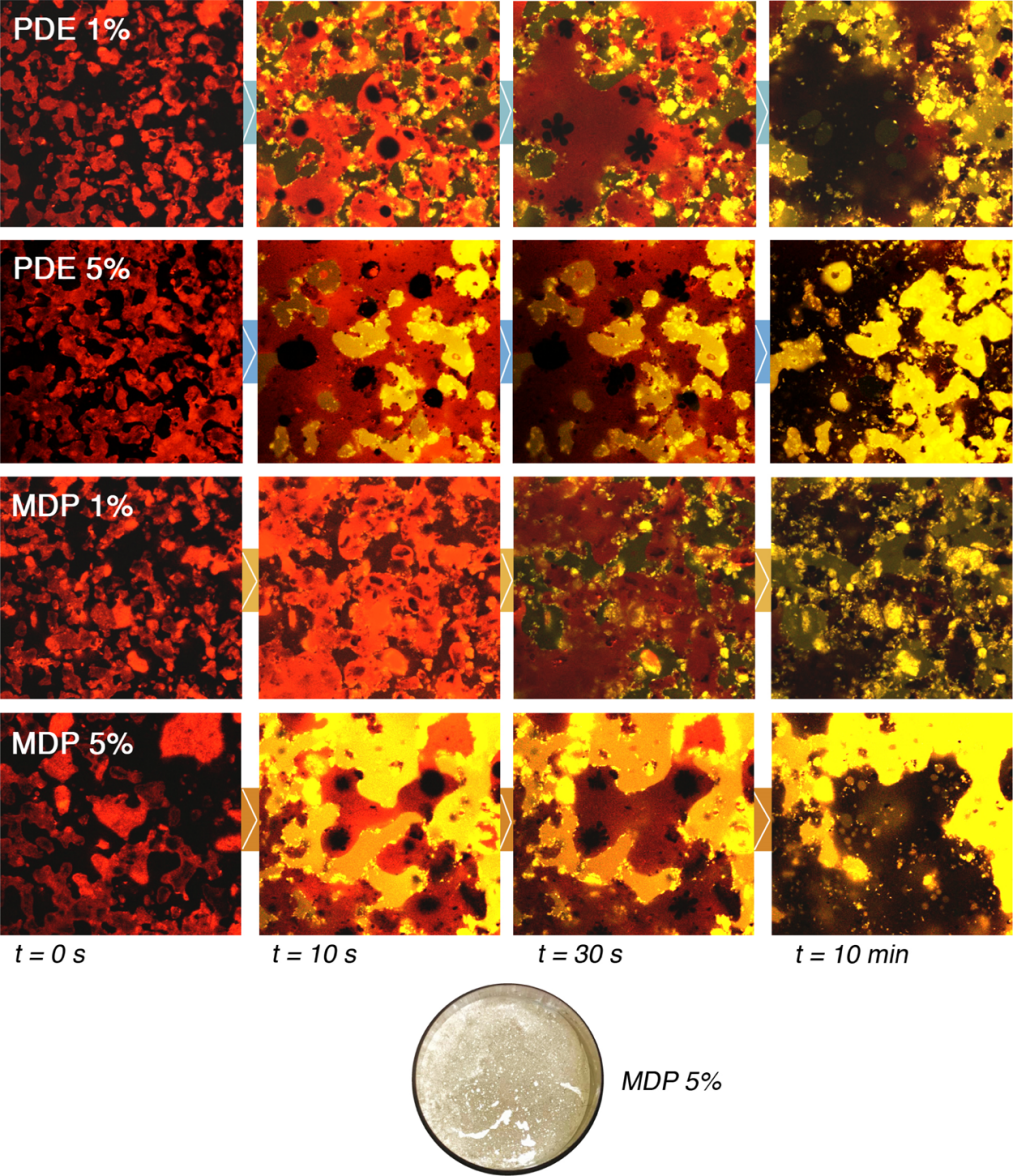
CLSM experiments on soil/NSF interaction. The round picture below the confocal images sequences represents the appearance of the glass incubated for 10 min with the MPD 5% micellar solution. Nile red fluorescence is seen as red; rhodamine 110 chloride fluorescence is seen as green; yellow areas indicate the co-presence of both fluorescent dyes. The bottom side of each CLSM frame is 150 μm long. In the bottom picture, cracks and holes are clearly visible as the result of the MPD 5% NSF action on the soil coating.

In order to follow the evolution of sample morphology at the glass/soil interface, as reported for CLSM experiments on soil/NSFs interactions (see Section 2.7.2), both the liquid aqueous phase and the soil layer were stained with fluorescent dyes. Figure 3 summarizes the result of an extensive CLSM investigation on several soiled glasses. In the false-colors images, soil is labeled with Nile red, whose fluorescence is seen as red, while rhodamine 110 chloride fluorescence is seen as green. The yellow areas indicate the co-presence of both fluorescent dyes and label the interaction of surfactant solution with soil. Because of the heterogeneous composition of the soil, the images reported in Figure 3 show patches with different colors, which evolve with time during the cleaning process. Four different 1 and 5% MPD and PDE solutions have been studied. As shown in Figure 3, the interaction of the surfactants with the soil is very fast in the first 30 s to 1 min. After this initial interaction, the soil morphology continues to evolve at slower rate. The appearance of the soil layer at the glass interface at *t* = 0 s shows the presence of several non-contact areas (dark zones), meaning that the adhesion (or wetting) of the soil to glass is not particularly favored, that is, the soil has a poor affinity for the glass slides surface, and in a few seconds, the oily phase present in the soil coalesces and rearranges itself in large droplets, recalling a dewetting-like process. This appears dark in the confocal images at *z* coordinates close to the glass slides surface. For both 5% MPD and PDE solutions, because of the presence of the dissolved rhodamine 110, the aqueous phase is initially seen as green, and turns to bright yellow when the Nile red, present in the soil layer, interacts (within 10 min of incubation) with rhodamine. At longer time of incubation, the oily phase of the soil is dark brownish because of the depletion in the fluorescent dye, which was initially dispersed in the coating. At the end of the cleaning process, the bright yellow spots unevenly distributed are related to starch and gelatin particles that remain adherent on the glass.

Even if the interpretation of the collected images is not straightforward, it is evident that surfactant concentration plays a major role in determining a displacement of the soil coating, by detaching it from the glass surface. As observed for polymer/NSF interactions, soil detachment from the surface may be regarded as the first key step of the removal process. The process observed for MPD 5% and PDE 5% is similar; however, on average, larger and more continuous soil detachment areas were evidenced in samples incubated with 5% MPD.

**Figure 4:**
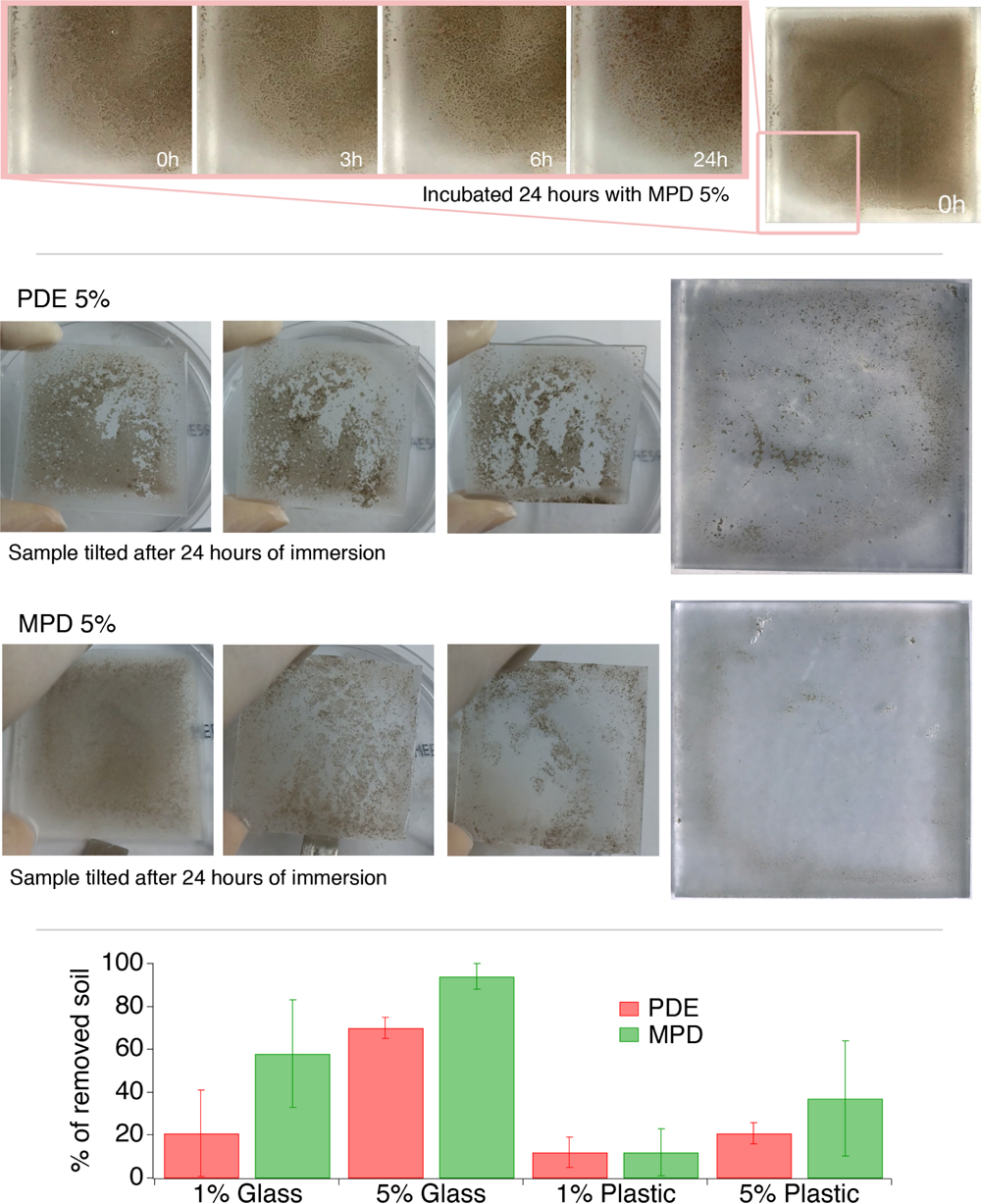
Soil removal experiments on glass slides. (Top) Sequence of zoomed picture taken during the 24 h of immersion of a soiled glass slide in the MPD 5% micellar solution. The dewetting-like process, with the formation of cracks and holes, is clearly visible. (Middle) Glass slides incubated respectively with PDE 5% and MPD 5% micellar solutions were tilted, in order to check for residual soil adhesion to the glass surface. The soil was partially (PDE) or completely (MPD) detached from the glass; the final appearance of treated glass slides is reported. (Bottom) The histogram shows the % of soil removal achieved with the different NSFs on the two different substrates, that is, glass and polystyrene. It is evident that soil removal from polystyrene is incomplete.

The effectiveness of MPD-based NSFs was also compared to PDE-based NSFs on macroscopic soil removal experiments performed on both frosted glass and polystyrene slides. Four NSFs (40 mL) used in CLSM experiments were left for 24 h in contact with the samples. The samples were monitored at 0, 3, 6, and 24 h. Figure 4-top shows that the majority of samples are unaffected by the action of the NSFs having PDE and MPD concentration below 5%. For 5% concentration, the dewetting-like process evidenced in CLSM experiments was clearly observable with the formation of cracks and holes in the originally coherent soil layer. The soil coating on polystyrene slides was adherent to the surface, and only in the case of the sample treated with PDE 5%, a significant (about 40%—see Figure 4-bottom) soil removal was observed. Figure 4-bottom shows that soil removal is proportional to surfactant concentration and that the MPD surfactant is the most efficient removing almost 100% for 5% MPD surfactant concentration in the absence of any mechanical action, see Figure 4-middle. This feature is very important in the conservation field in view of soil removal from the delicate and fragile surface of works of art.

To better clarify the MPD and PDE performances, the contact angle for pure water on soil was measured. The contact angle at the water/soil interface was 52 ± 8°. After the artificial soil immersion for 1 min in the two 1% surfactant solutions, the contact angle was 29 ± 1 and <10° for soil incubated with MPD and PDE, respectively. This, contrarily to what can be expected, results in a lower effectiveness of MPD surfactant, when a solubilization process is involved in the cleaning mechanism. SAXS measurements performed on the cleaning fluids samples at 0, 3, 6, and 24 h on glass and polystyrene slides, shed light on the different cleaning mechanism for the two surfactants.

**Figure 5:**
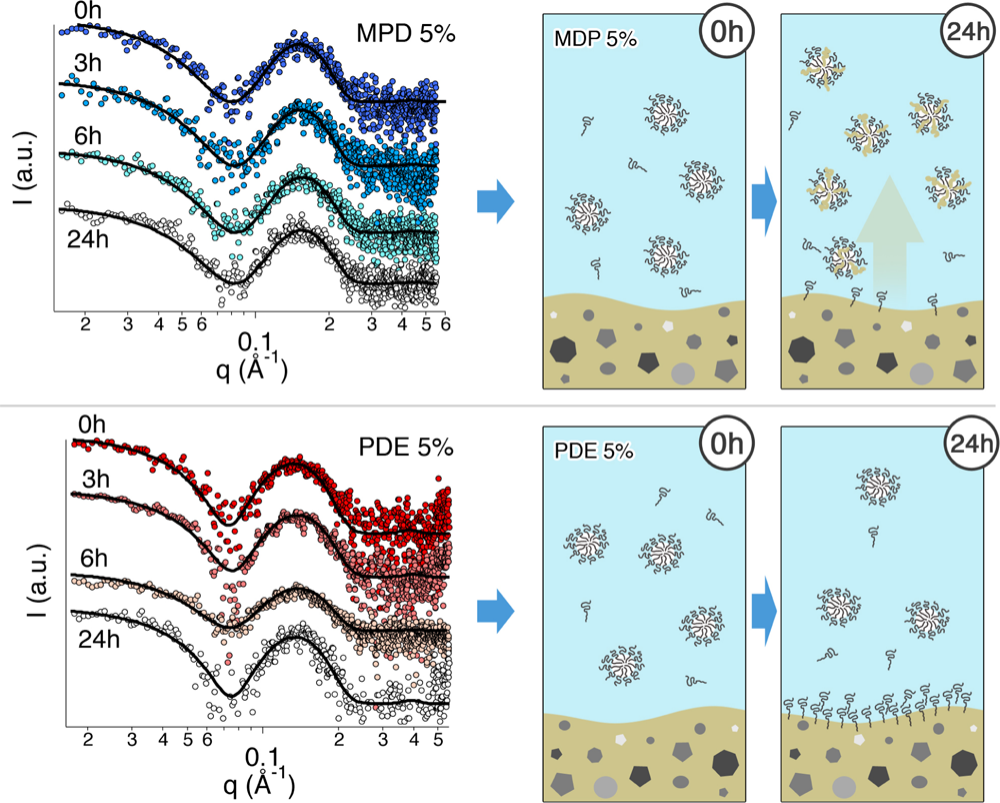
SAXS curves of the MPD 5% and PDE 5% systems, interacting with soiled glass slides. The measurements were performed on samples taken at different times, that is, 0, 3, 6, and 24 h. Solid black lines represent the best fitting curves for experimental data. The curves have been offset for sake of clarity.

Figure 5 reports the scattering curves of 5% MPD and PDE solutions in contact with soiled glass slides for 24 h. The main fitting results are listed in Table 5, where the volume fraction and the micelles core radius and shell thickness are reported (the description of the fitting model is reported in Supporting Information). According to published data on the effective volume fraction of these two surfactants in water,^46^ it was assumed that the volume fraction of both MPD and PDE 5% w/w (at *t* = 0 h) is 0.2 with a 10% uncertainty on this value.

**Table 5:** SAXS Fitting Results^a^

			sampling time			
	system	fitting parameter	0 h	3 h	6 h	24 h
soiled glass	MPD 5%	ϕ	0.20 ± 0.02	0.20 ± 0.02	0.33 ± 0.03	0.36 ± 0.02
		*r*_c_ (Å)	14.6 ± 1.2	14.5 ± 0.1	13.9 ± 0.9	13.9 ± 0.9
		*t* (Å)	19.0 ± 1.7	19.0 ± 0.3	19.0 ± 1.6	19.2 ± 1.6
	PDE 5%	ϕ	0.20 ± 0.02	0.25 ± 0.03	0.17 ± 0.02	0.15 ± 0.01
		*r*_c_ (Å)	15.6 ± 0.1	15.1 ± 0.1	15.2 ± 0.1	15.3 ± 0.2
		*t* (Å)	23.5 ± 0.2	22.4 ± 0.2	21.7 ± 0.2	23.4 ± 0.3
soiled polystyrene	MPD 5%	Φ	0.20 ± 0.02	0.13 ± 0.02	0.15 ± 0.02	0.14 ± 0.01
		*r*_c_ (Å)	14.6 ± 1.2	15.7 ± 0.1	15.7 ± 0.1	14.7 ± 0.5
		*t* (Å)	19.0 ± 1.7	22.0 ± 1.4	22.0 ± 0.3	20.0 ± 1.2
	PDE 5%	ϕ	0.20 ± 0.02	0.17 ± 0.01	0.14 ± 0.01	0.13 ± 0.01
		*r*_c_ (Å)	15.6 ± 0.1	15.3 ± 0.2	15.5 ± 0.4	15.7 ± 0.7
		*t* (Å)	23.5 ± 0.2	22.0 ± 0.3	23.0 ± 0.2	23.0 ± 1.1

a ϕ is the volume fraction of the scattering objects, with respect to the whole volume system; *r*_c_ is the average core radius; *t* is the shell thickness.

The geometrical parameters obtained from the fitting are in good agreement with previously published SAXS data on these surfactants, where a model-free Fourier-transform approach was used.^46^ It is worth noting that the shell thickness is significantly high for both surfactants, having MPD micelles a smaller shell, in agreement with literature data,^46^ that report a lower hydration number for the polar head of MPD because of the methyl capping at the end of the polyoxyethylene chain. The results obtained for MPD and PDE micellar solutions in contact with the soil layer show a different behavior for the two surfactants. For both surfactants, micelles’ size is almost constant, while the volume fraction significantly changes. For the 5% MPD solution, the volume fraction of scattering particles starts to increase after 6 h of interaction with the soil layer, and after 24 h, it is about 80% larger than its original value. SAXS data show that micelles do not grow indicating that the solubilization into the micelles of hydrophobic components from the soil layer occurs with a subsequent reorganization of the micellar structure. In other words, the solubilization of soil leads to a higher number of micelles with oil molecules replacing the surfactant, with the resulting effect of the presence of aggregates with similar size of the original micelles but with a different number (see the top cartoon in Figure 5).

Considering the composition of the artificial soil, see Table S3, it can be assumed that main soil components solubilized in the micelles come from mineral oil and olive oil. The mineral oil present in the artificial soil is mainly composed of saturated linear C_15_–C_50_ hydrocarbons, while the main component of olive oil is glyceryl trioleate^57^ or triolein, a bulky and high molecular weight triglyceride. Several studies in the literature, about the solubilization of hydrophobic substances by nonionic surfactants’ micelles,^57−61^ are consistent with the interaction mechanism between MPD micelles and the soil. In particular, Kralchevsky et al. proposed a mechanism for the solubilization of triolein into nonionic micelles, where a direct interaction of the surfactant micelles with the interface, accompanied by an uptake of oil, occurs.^61^ Interestingly, they found that after triolein solubilization, the rod-like micelles did not swell but rather they split into several smaller micelles, undergoing a structural reorganization,^61^ similarly to the SAXS results of the present study. Interestingly, 5% PDE solution shows the opposite trend for the volume fraction of scattering objects. In fact, after an initial slight size increase, the volume fraction decreases and after 24 h is about 25% less than its original value. Therefore, at the end of the process, a number of micelles had disappeared because of surfactant depletion from the aqueous phase as a consequence of significant PDE adsorption on the soil surface (see the bottom cartoon in Figure 5). These results clearly account for the higher effectiveness of MPD in removing the soil from glass surfaces, even in the absence of any mechanical action.

**Figure 6:**
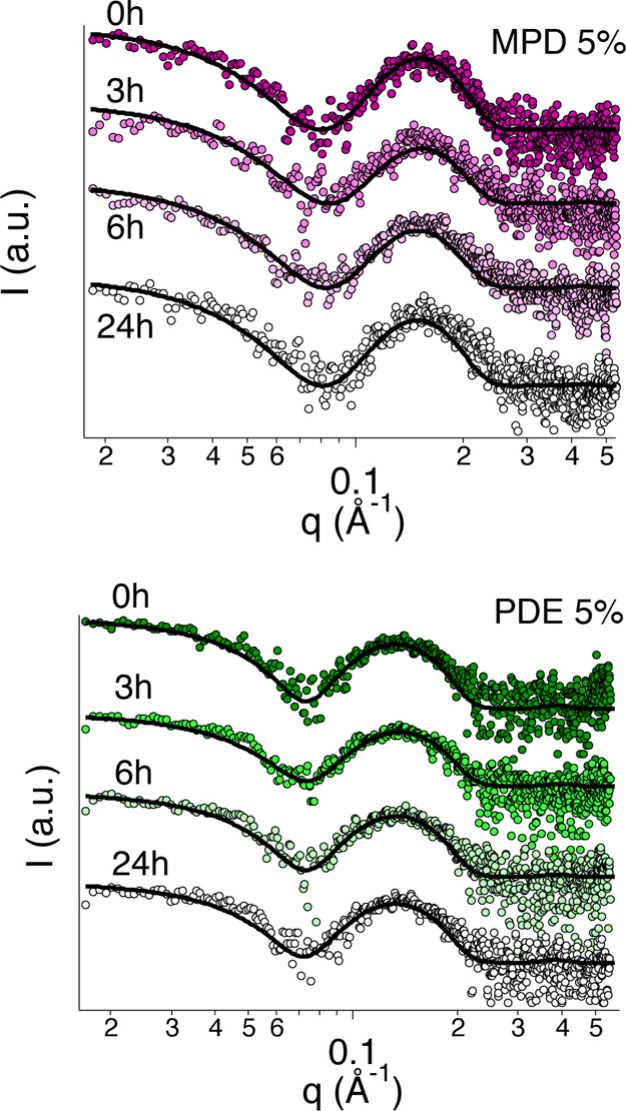
SAXS curves of the MPD 5% and PDE 5% systems, interacting with soiled polystyrene slides. The measurements were performed on samples taken at different times, that is, 0, 3, 6, and 24 h. Solid black lines represent the best fitting curves for experimental data. The curves have been arbitrarily stacked for sake of clarity.

**Figure 7:**
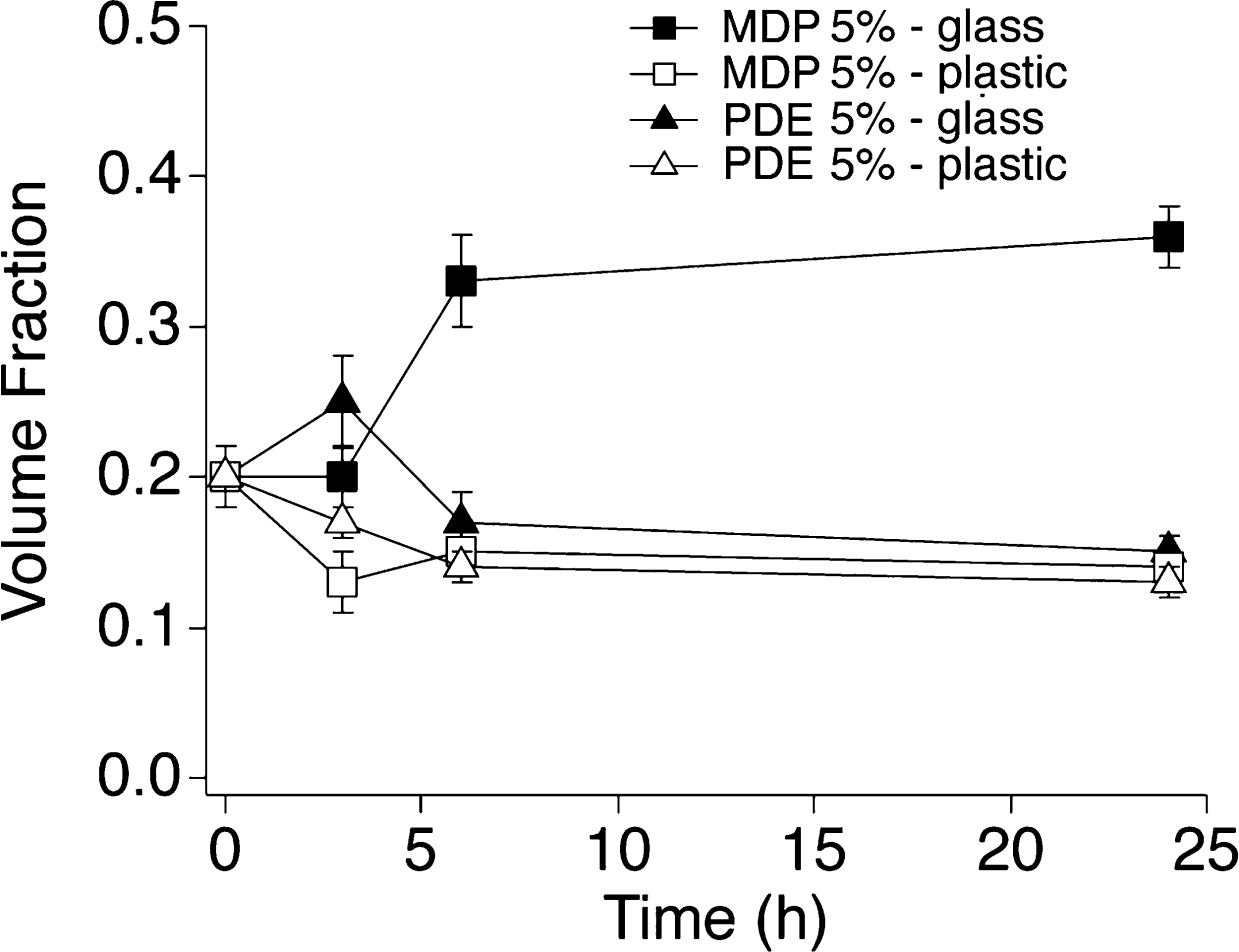
Trend of the volume fraction of the scattering objects for the 5% surfactant systems interacting with glass and polystyrene slides for 24 h, as obtained by the analysis of SAXS data.

SAXS from 5% MPD and PDE aqueous solutions interacting with soil layers on polystyrene slides shows a different behavior with respect to glass slides, which is mainly due to the different hydrophilic character of the two materials. Figure 6 reports the SAXS curves, together with their best fitting. The main results are listed in the second half of Table 5. Micelles’ size is almost unaltered after the surfactant interaction with soil, and the volume fraction decreases for both surfactants, similarly to PDE interacting with the soiled glass slide. This is related to the higher affinity between polystyrene and the soil layer that inhibits the solubilization of its oily fraction into the micellar core. Thus, because of the adsorption of surfactant at the soil surface, a fraction of micelles is disrupted, with a subsequent decrease in the volume fraction of scattering objects. Figure 7 shows the trend of the volume fraction in the different cases, highlighting that the oil solubilization occurs only in the case of MPD 5% interacting with soiled glass.

The above results report a detailed picture on the surfactant interactions with two different “coatings” commonly found in classic and contemporary/modern art. Overall, it is shown that a tiny change in the molecular structure of the PDE leads to consistent changes in the mechanisms of action, the kinetics, and the cleaning efficacy of the surfactant.

## 4 Conclusions

Complex systems composed by MPD have been investigated, and its effectiveness was compared to PDE, a conventional nonionic amphiphile, for the cleaning of two common materials disfiguring the aesthetical aspects of works of art. In particular, MPD- and PDE-NSFs were challenged for the removal of poly(ethyl methacrylate/methyl acrylate) 70:30, p(EMA/MA), commercially known as Paraloid B72 from glass and polystyrene surfaces, while aqueous micellar solutions of the two surfactants were used for the cleaning of artificially soiled surfaces. The overall results highlighted the better performance of MPD both for the polymer and the soil removal from coated surfaces. The interaction mechanism of NSFs for the removal of p(EMA/MA) polymer, observed at the micro-scale through CLSM imaging, involves a dewetting-like process. The polymer is detached from the surface and coalesces into separated droplets as the liquid phase/solid surface interfacial area increases. The PDE- and MPD-NSFs exhibit different mechanisms that depend both on the organic solvent and surfactant because of the different surface tensions and to the different adsorption/penetration of MPD onto/into the polymer film, with respect to PDE. This is likely due to the methyl capping of the surfactant polar head and to the presence of the ester group between the hydrophilic and hydrophobic moiety of the surfactant molecule (PDE). FCS provided a more detailed picture of the cleaning process showing that the surfactants present in the NSFs are able to penetrate through the Paraloid B72 film, that acts as a sort of “sieve”, and reach the polymer/solid surface interface, where liquid-filled cavities are formed. Moreover, CLSM experiments highlighted better performances of MPD, if compared to PDE, also in soil removal. The mechanism involves a dewetting-like process, where the oily phase is detached from the glass or polystyrene substrates and coalesces into large droplets. Surfactant concentration was found to be crucial to boost the interaction with the heterogeneous soil. 1% surfactant solutions are less effective than 5%, even if micelles are present in both cases. Differently to PDE that adsorb on the soil layer surface it was found, for both glass and polystyrene substrates, that MPD micellar solutions solubilize soil. Both surfactants allow the removal of soil and grime with different efficacy, no mechanical action, and with different times. The time necessary to perform the cleaning and the mechanical action in conservation are of uppermost importance because long application times and mechanical action should be avoided particularly in the case of fragile and delicate surfaces as those of works of art, which hardly tolerate mechanical stresses during the cleaning operations. Overall, the results reported in the present work open up to new formulations for better-performing and safer cleaning systems to be used by restorers for the conservation of cultural heritage or in other applications as detergency, cosmetics, and so forth.

## Supporting Information Available

Details on materials and methods and additional figures of all FCS curves, cleaning tests, and surface tension measurements (PDF)

## Notes

Michele Baglioni and Piero Baglioni are not related.
