# Supplementary material for: Nonionic Surfactants for the Cleaning of Works of Art: Insights on Acrylic Polymer Films Dewetting and Artificial Soil Removal
Source: ACS Appl Mater Interfaces. 2020 May 12;12(23):26704–16. doi: 10.1021/acsami.0c06425 (PMC8007071; doi:10.1021/acsami.0c06425)
Supplement: Supplementary file 1 — am0c06425_si_001.pdf [file am0c06425_si_001.pdf]

Supporting Information for

NONIONIC SURFACTANTS FOR THE CLEANING OF WORKS OF ART: INSIGHTS ON ACRYLIC POLYMER FILMS DEWETTING AND ARTIFICIAL SOIL REMOVAL

Michele Baglioni[a°], Teresa Guaragnone[a], Rosangela Mastrangelo[a], Felipe Hidetomo Sekine[b], Taku Ogura[b,c,d,] Piero Baglioni[a°*]

a Department of Chemistry and CSGI, University of Florence, via della Lastruccia, 3, Sesto Fiorentino, Florence, 50019, Italy;
b NIKKOL GROUP Nikko Chemicals Co., Ltd., 1-4-8, Nihonbashi-Bakurocho, Chuo-ku, 103-0002, Tokyo, Japan;
c NIKKOL GROUP Cosmos Technical Center Co., Ltd., 3-24-3 Hasune, Itabashi-ku, 174-0046, Tokyo, Japan;
d Research Institute for Science & Technology, Tokyo University of Science, 2641, Yamazaki, Noda-shi, Chiba 278-8510, Japan.

*Corresponding author
Piero Baglioni; e-mail: baglioni@csgi.unifi.it

°No kinship exists among these authors.

Conflict of interest: none

Additional details on Materials and Methods

NSFs

Table S1. NSFs used in CLSM experiments on polymer removal. Amounts are reported as %(w/w)

PC				MEK			
**MPD**		**PDE**		**MPD**		**PDE**	
H_2O	79.9	H_2O	79.9	H_2O	83.8	H_2O	83.8
PC	15.1	PC	15.1	MEK	11.2	MEK	11.2
MPD	5	PDE	5	MPD	5	PDE	5

Note that water/solvent molar ratio was kept constant and equal to 30:1 in each NSF.

Table S2. Composition of NSFs used in polymer removal tests on glass slides.

Component	% (w/w)
Water	60
Surfactant[a]	~3[b]
BuOH	10
PC	8
EtAc	8
MEK	11

[a]Each of the 4 NSFs tested included a different surfactant selected from this list: MPD, PDE, SDS, DDAO. [b]The amount of surfactant was calculated according to each molecular weight, in order to have the same moles of each amphiphile in the different systems.

Artificial soil

Table S3. Artificial soil composition.

Soil component	Amounts[a]
Carbon black	2.0 g
Iron oxide	0.5 g
Silica	1.7 g
Kaolin	20.0 g
Gelatin powder	10.0 g
Soluble starch	10.0 g
Cement	17.5 g
Olive oil	10.0 ml
Mineral oil	20.0 ml

[a]The amounts here reported are relative to 1 L of dispersion in white spirit.

The solid components were weighed and mixed together. Then, olive oil and mineral oil were added to obtain a viscous paste, which was diluted with white spirit, in order to obtain a solution/dispersion that could be applied on the samples surface. After complete evaporation of white spirit, an uneven brownish oily coating remains on treated surfaces.

SAXS fitting model

MPD and PDE micelles, either in just water or in water/solvent mixtures, were modeled as core-shell monodisperse spheres, thus defined by two contrasts, i.e. bulk/shell and shell/core. Each volume region (i.e. bulk, shell and core) is characterized by a scattering length density (SLD), ρ_{bulk}, ρ_{shell} and ρ_{core}. For general globular micelles of homogeneous scattering length density, the total scattered intensity I(q) (cm^{-1}) is given by:[1,2]

$$I(q) = N_P V_P^2 \Delta\rho^2 P(q) S(q) + \mathrm{bkg}_{\mathrm{inc}}$$

where N_p is the number density of the scattering objects (cm^{-3}), V_p is the their volume (cm^3), $\Delta\rho$ is the contrast term (cm^{-2}), $P(q)$ is the form factor and $S(Q)$ is the structure factor. MPD and PDE are nonionic surfactants, i.e., their micelles are uncharged, and concentrations used in this study (up to 5% w/w) should grant that systems are diluted enough to make intermicellar interactions negligible. Thus, in our model, $S(q) = 1$, and the scattering intensity of a monodisperse spherical particle with a core-shell structure was calculated as follows:[3]

$$I(q) = \frac{K\phi}{V_P}\left[\frac{3V_c(\rho_{core} - \rho_{shell})F(qr_c)}{qr_c} + \frac{3V_P(\rho_{shell} - \rho_{bulk})F(qr_P)}{qr_P}\right]^2 + bkg$$

where K is a scaling factor (due to the fact that our data are not reported in absolute intensity); ϕ is the volume fraction of the scattering objects, V_P and V_c are the volume of the whole particle and of the sole core, respectively; the total radius of the particle, r_P, is given by the sum of the core radius, r_c, and the shell thickness, t, i.e., $r_P = r_c + t$; and the $F(x)$ function is defined as follows:

$$F(x) = \frac{(\sin x - x\cos x)}{x^2}$$

The model used to fit the ternary NSFs, including PC and MEK, slightly differs from the one just described in that it takes into account a polydisperse core, which follows the Schultz distribution. The form factor is then normalized by the average particle volume:

$$\langle V \rangle = \frac{4\pi}{3}\langle r_c^3 \rangle$$

where:

$$\langle r_c^3 \rangle = \frac{(z+3)(z+2)}{(z+1)^2}\langle r_c \rangle$$

and z is the width parameter of the Schultz distribution:

$$z = \frac{1}{\left(\frac{\sigma}{\langle r_c \rangle}\right)^2} - 1$$

being σ^2 the variance of the distribution. The polydispersity index, PDI, reported in Table 2 is then defined as $\sigma/\langle r_c \rangle$, and its value is comprised between 0 and 1.

FCS data analysis

The curves shown were normalized and averaged (10 repetitions per sample in the case of fluids before the interaction; only 6 repetitions per sample in the cavities at the polymer/glass interface or in the dewetted polymer, to avoid further uncontrolled evolution of the system). An aqueous solution of Alexa Fluor 568 dye was used for FCS calibration.[4]

The autocorrelation function G(t) obtained by FCS measurements can be analyzed assuming that the fluorescent species is diffusing across a 3D-ellipsoidal Gaussian volume and that the three-dimensional Brownian diffusion is the only phenomenon influencing the decay. All the decays analyzed in this paper are two-components decays. In this case, G(t) can be fitted according to:[5]

$$G(\tau) = \frac{1}{N}\left[f_1\left(1 + \frac{\tau}{\tau_{D1}}\right)^{-1}\left(1 + \frac{\tau}{S^2\tau_{D1}}\right)^{-1/2} + (1 - f_1)\left(1 + \frac{\tau}{\tau_{D2}}\right)^{-1}\left(1 + \frac{\tau}{S^2\tau_{D2}}\right)^{-1/2}\right]$$

where N is the average number of fluorescent molecules detected inside the confocal volume ($N = CV$, with $V = \pi^{3/2}{w_0}^3 S$ and C the concentration), f_1 is the percentage of the contribution of $\tau_{D,1}$ to the total decay time, $\tau_{D,i}$ are the decay times, and $S = z_0/w_0$ is the ratio between the axial and the lateral dimensions of the confocal volume, determined through the calibration procedure with Alexa 568. The diffusion coefficients D_i of the fluorescent probe can be determined considering that:

$$\tau_{D,i} = \frac{{w_0}^2}{4D_i}$$

Additional figures

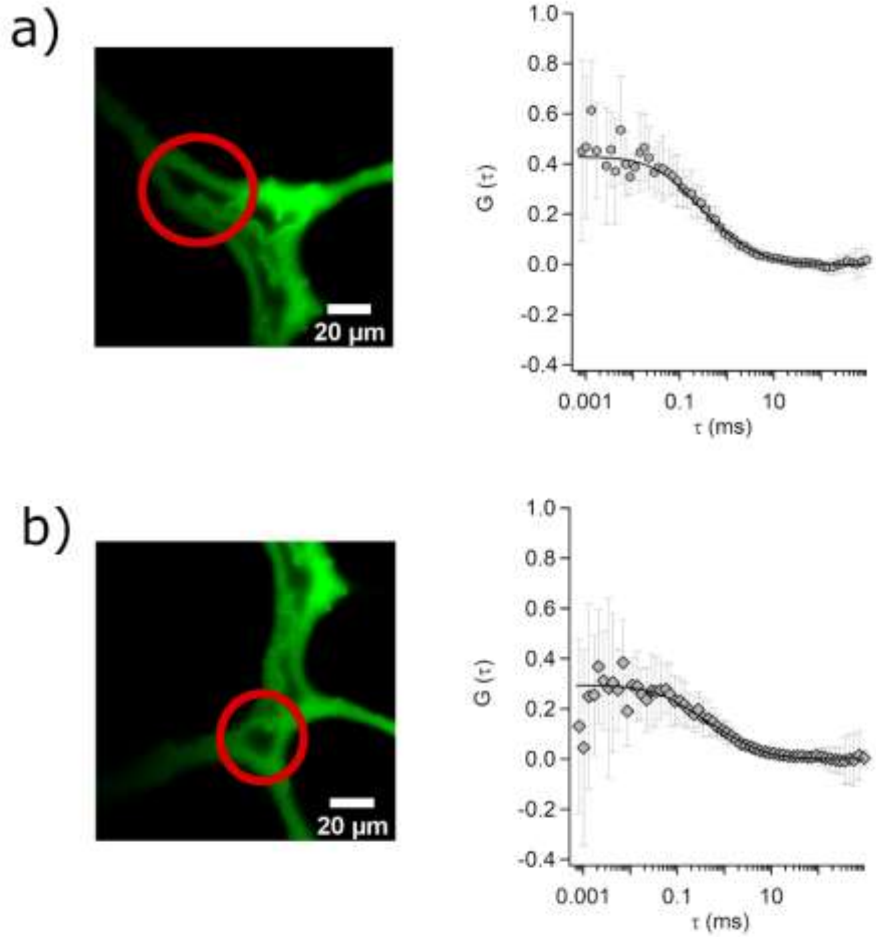

Figure S1. Left (a-b): Cavities formed at the glass-polymer film interface after 5 min (a) and 15 min (b) of interaction with H2O/MEK/MPD; the red circles show the cavity where the diffusion of Bodipy-labeled micelles was measured. Right (a-b): Averaged FCS curves (markers), obtained probing the circled areas, and two-components decay fitting (solid lines).

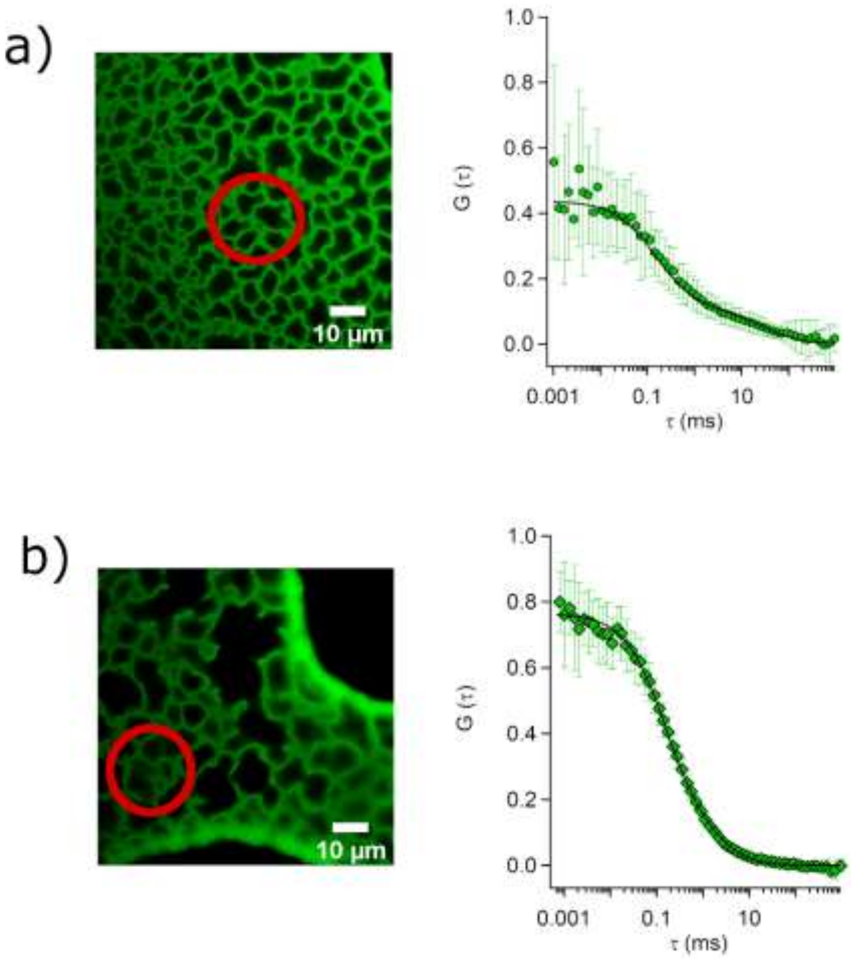

Figure S2. Left (a-b): Cavities formed at the glass-polymer film interface after 5 min (a) and 15 min (b) of interaction with H2O/MEK/PDE; the red circles show the cavity where the diffusion of Bodipy-labeled micelles was measured. Right (a-b): Averaged FCS curves (markers), obtained probing the circled areas, and two-components decay fitting (solid lines).

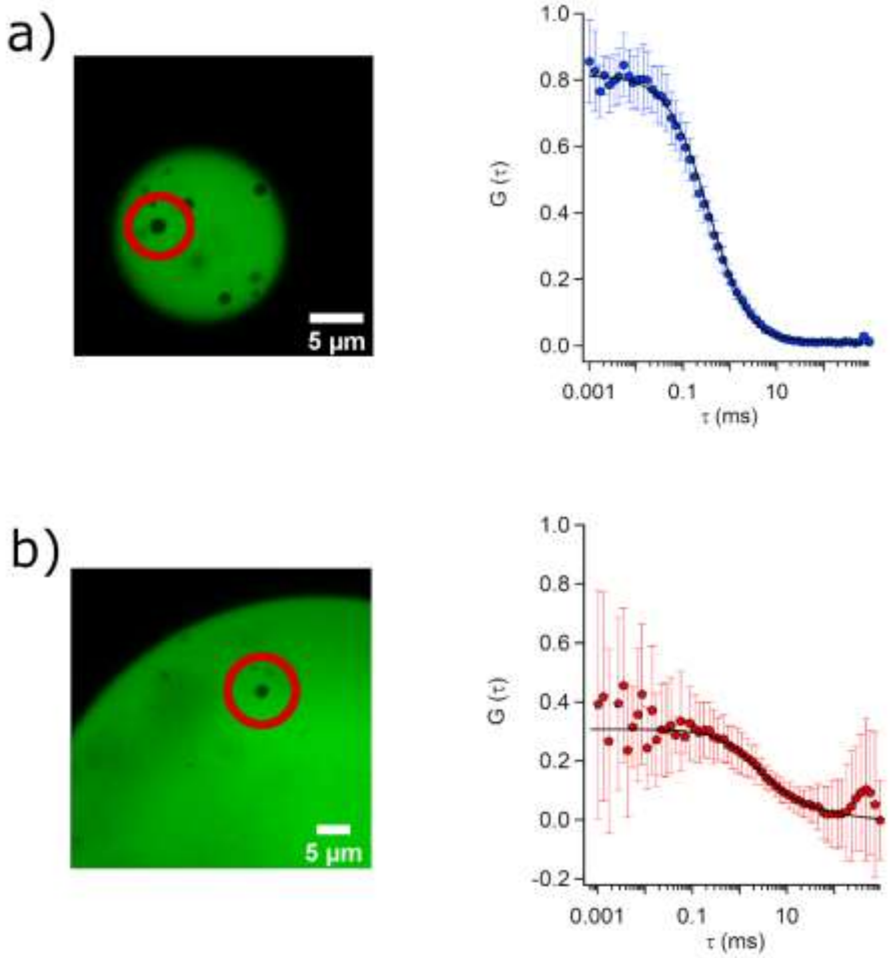

Figure S3. Left (a-b): Cavities formed inside dewetted polymer blobs for (a) H2O/PC/PDE and (b) H2O/PC/MPD after few minutes of NSFs-film interaction; the red circles show the cavity where the diffusion of Bodipy-labeled micelles was measured. Right (a-b): Averaged FCS curves (markers), obtained probing the circled areas, and two-components decay fitting (solid lines).

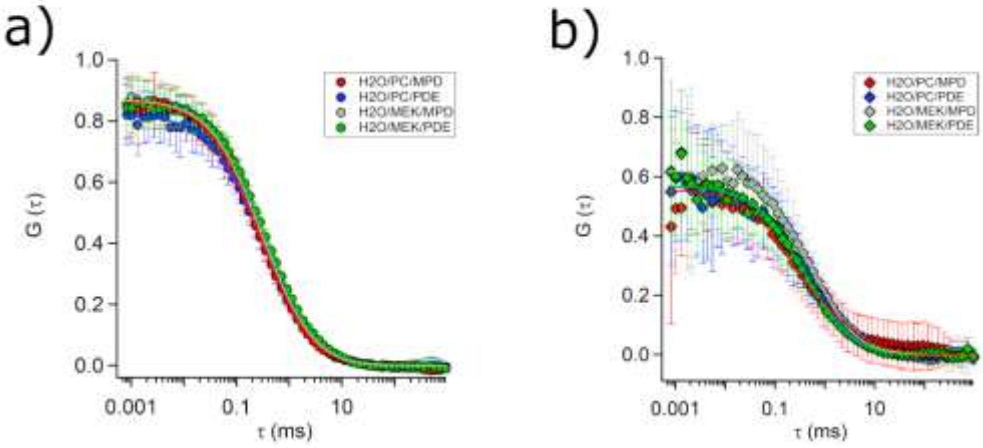

Figure S4. Averaged FCS curves (markers) and two-components decay fittings (solid lines) for the NSFs before (a) and after 20 min of interaction (b) with the polymer film.

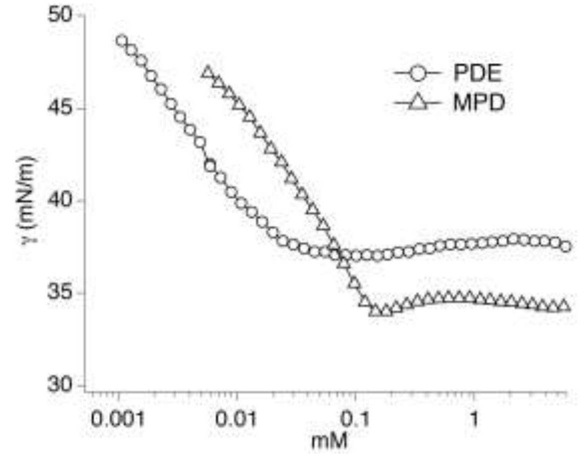

Figure S5. Surface tension of MPD and PDE aqueous solutions, as a function of surfactant concentration.

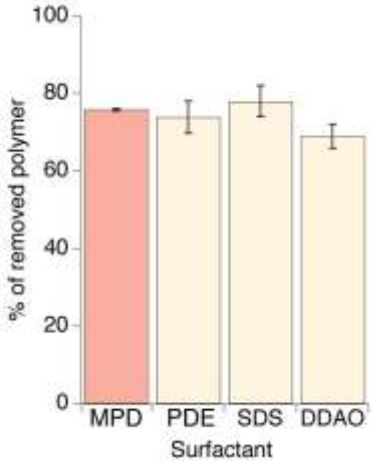

Figure S6. Histogram showing the results of polymer tests performed on glass slides with 4 NSFs differing from each other only in the surfactant.

References

(1) Sheu, E. Y.; Chen, S. H. Thermodynamic Analysis of Polydispersity in Ionic Micellar Systems and Its Effect on Small-Angle Neutron Scattering Data Treatment. *J. Phys. Chem.* **1988**, *92* (15), 4466–4474. https://doi.org/10.1021/j100326a044.

(2) Liu, Y. C.; Ku, C. Y.; LoNostro, P.; Chen, S. H. Ion Correlations in a Micellar Solution Studied by Small-Angle Neutron and x-Ray Scattering. *Phys. Rev. E* **1995**, *51* (5), 4598–4607. https://doi.org/10.1103/PhysRevE.51.4598.

(3) Guinier, A.; Fournet, G. *Small-Angle Scattering of X-Rays*; Wiley, 1955.

(4) Montis, C.; Maiolo, D.; Alessandri, I.; Bergese, P.; Berti, D. Interaction of Nanoparticles with Lipid Membranes: A Multiscale Perspective. *Nanoscale* **2014**, *6* (12), 6452–6457. https://doi.org/10.1039/c4nr00838c.

(5) Milani, S.; Baldelli Bombelli, F.; Pitek, A. S.; Dawson, K. A.; Rädler, J. Reversible versus Irreversible Binding of Transferrin to Polystyrene Nanoparticles: Soft and Hard Corona. *ACS Nano* **2012**, *6* (3), 2532–2541. https://doi.org/10.1021/nn204951s.
